# London’s fatbergs and affective infrastructuring

**DOI:** 10.1177/0306312720917754

**Published:** 2020-05-01

**Authors:** Mike Michael

**Affiliations:** Department of Sociology, Philosophy and Anthropology, University of Exeter, UK

**Keywords:** affect, fatbergs, infrastructure, infrastructuring, publics

## Abstract

This exploratory article considers the accumulations of fat and other materials in London’s sewerage system – known as fatbergs in the UK – in terms of the processes of infrastructuring. In particular, drawing on a range of media, including a major museum exhibition, numerous newspaper and online articles, and a TV documentary, this article analyses how London’s fatbergs have been affectively enacted. The affects identified include: disgust in the composition of the fatberg, pride in the London-ness of the fatbergs, admiration at the ‘flushers’ courage, generic horror at the sewers, shame in the flushing of wet wipes, and anxiety about microbial threats. Such enactments simultaneously perform the fatbergs, the sewerage infrastructure, and the public audiences, through what we can call ‘affective infrastructuring’. This extends the analysis of infrastructuring to encompass the ways in which public audiences are affectively ‘made’. The article also suggests that the various affective enactments of the fatberg cumulatively perform London as spatially uniform and the sewerage system as temporally naturalized. A critical implication of this is an effacement of, on the one hand, class and cultural difference and, on the other, historical specificity.

And so, ladies and gentlemen, as the toilet of time washes away the wet wipe of wastefulness through the u-bend of ubiquity along the sewer pipe of posterity and into the fatberg of fate, I notice it’s the end of the show.

Jack Dee, I’m Sorry I Haven’t a Clue, BBC Radio 4 (2 July 2018)

In September 2017 a fatberg weighing around 130 tonnes with a length of over 250 metres was discovered in a Whitechapel sewer. An initial reading of the media reports on this London fatberg might give the impression that the feelings it precipitated were mainly ones of horror and disgust. With the exposure of the fatberg there seemed to be an intersection and an interplay of a variety of excreta and effluvia, of human wastes and weaknesses, that suddenly came to light. But on closer reading, the enactments of the fatberg invited other affects, including pride, admiration, even pleasure. What roles might this variety of affects be playing? In particular, how do these affects serve in the ‘doing’ of an infrastructure such as London’s sewerage system? More broadly, does this case facilitate an account of the place of affect in infrastructuring?

This exploratory article concerns the ways in which London’s fatbergs –accumulations of fat and other materials in London’s sewerage system – have been multiply enacted across a range of media, including a major museum exhibition, numerous newspaper and online articles, and a TV documentary. What emerges is a series of narratives, images and themes that serve to perform a range of fatbergs and their associated affects. This is analysed in terms of infrastructuring, or, more specifically, affective infrastructuring, in which particular public affects are associated with London’s fatbergs and the sewerage system in which they are found. The various fatberg-infrastructuring-affect links also depict a singular London, sewerage system, fatberg and public audience. The critical implication of this uniforming is that, on the one hand, class and cultural difference are geographically effaced, and, on the other, the sewerage system is historically decontextualized, that is to say, naturalized.

## Infrastructure and affect

STS’s engagement with infrastructure has been a long and complex one. Infrastructure has been analysed in terms of, for example: its sociomaterial prehistory and its contemporary invisibility (e.g. Bowker and Star, 1999; Star, 1999; Star and Ruhleder, 1996), its constitutive heterogeneity and its ontological experimentality (e.g. Bowker et al., 2010; Bruun Jensen, 2015; Bruun Jensen and Morita, 2015), its status as a manifestation of enchanting promise and a site of continuous, disparate practices of maintenance (e.g. Blok et al., 2016; Harvey and Knox, 2012), an absence of singular solutions and an exercise in strategic ignorance (Anand, 2015; Edwards et al., 2009), a topology of relations and the verb form ‘infrastructuring’ (e.g. Harvey, 2012; Karasti and Blomberg, 2018).

What is clear across these various treatments is that infrastructure is a multiplicity of relations in need of continuous maintenance, functions in a variety of ways encompassing a range of practitioners and users, and, as such, is not uncommonly uncertain in its operations. In the context of the London fatberg, a number of initial observations can be made.

First, prima facie it appears that the fatbergs are failures of London’s sewerage system. This suggests that it is problematic to address fatbergs as if they are themselves infrastructures. That is true enough. However, fatbergs are indicative of the functioning of infrastructures, or rather, of the processes by which infrastructuring is conducted. Fatbergs are thus putative problems for the sewerage system and its operation, and because of this, as various authors have long noted (e.g. Star, 1999), fatbergs are a medium through which to explore that system and its operation. Taking such infrastructures as multiple and subject to continuous maintenance – that is, adopting a framing that emphasizes the dynamic processes of infrastructuring – suggests that problems (such as those posed by fatbergs) can facilitate an analysis of the multiple factors entailed in infrastructuring. In the case of the fatberg, or so I will argue, particular elements that need to be addressed in order to maintain the infrastructure are the ‘audiences’ of the sewerage system. These, too, are part of diverse elements that comprise the sewerage system. Were these audiences to lose heart in the system, were their expectations of a ‘working system’ to shift negatively, then the infrastructure would be compromised. As discussed below, a key way in which the ‘working-ness’ of the system is sustained is through affective means – by shaping the affects of those audiences in which a problem such as a fatberg becomes, in one way or another, ‘acceptable’, ‘ignorable’ or ‘ironicized’.

Second, publics become aware of the fatbergs not through their direct impact, but in terms of the threats they pose as enacted by various spokespersons, across a variety of media (news, documentary, and museum). In this respect, they differ from more ‘directly’ encountered infrastructural impacts. Examples of such ‘direct’ encounters with the effects of infrastructures include: observations of grand infrastructural projects that precipitate a feeling of ‘technological sublime’ (Nye, 1994), the mundane corporeal sense of comfort shaped by lighting and heating systems and standards (Shove, 2003), the experiences of discomfort that arise with the pre- and pro-scriptions of, say, transport or building infrastructures (Akrich, 1992; Akrich and Latour, 1992; Latour, 1991, 1992), and the everyday sociomaterial engagements with infrastructures (such as roads) that inflect with wider political processes (Knox, 2017). Of course, all these encounters are also mediated – that is, shaped by various representations (e.g. Michael, 2000). In the case of the fatberg, public audiences access it entirely indirectly through various forms of mediated representation.

Third, and related, the enactment of the fatbergs is, as mentioned above, notably affective. To be sure, London’s sewerage infrastructure is routinely represented as system on the verge of collapse. A typical refrain is that Joseph William Bazalgette’s brilliant design (see Cook, 2001) which was originally supposed to serve a population of 2.5 million people, now serves around 9 million, and Londoners are at risk from all sorts of unpleasant (back)flows of sewage. Hope lies in the new £5 billion ‘super sewer’ (O’Grady, 2018). In this context, the fatberg is enacted in ways that point to current dangers posed by an infrastructure that faces unforeseen challenges, not least those posed by the combination of fat and wet wipes. As we shall see, however, the affects that are attached to such enactments of the fatberg turn out to be a complex mixture of the positive and the negative.

Fourth, infrastructures, in that they are constitutively heterogeneous, do not address a singular function, are not fully knowable, and do not function seamlessly or predictably. In one STS terminology, they entail an ‘ecology of practices’ (Blok et al., 2016; Stengers, 2005) spanning many specialisms and interests, all vying to address different conceptualizations of the problems that the infrastructure is supposed to solve (does a sewerage system remove waste in the here and now as efficiently as possible, develop capacity for future population growth and environmental changes such as shifts in tidal patterns, protect the ‘flushers’ that work in the sewers, turn a profit, ensure adaptability to new types of waste, compare favourably to the systems of other major cities?). This multiplicity also emerges in relation to the fatbergs and their associated affects: the various enactments of the London fatbergs invited a panoply of feelings and emotions . For instance, the fatbergs were indicative, at once, of a unique manifestation of particular patterns of consumption and waste disposal, of health-endangering by-products of an infrastructure that is barely fit for its purposes, or of a disgusting amalgam of ‘natural’ and ‘unnatural’ bodily excreta. Ironically, as we suggest below, this multiplicity arguably congeals to perform a singular fatberg, along with a singular London and singular public audience.

Fifth, it is worth emphasizing that if infrastructures are not ‘objects’ but sets of heterogeneous relations in need of continuous maintenance and repair, it might be the case that part and parcel of this is the infrastructuring of audiences. That is to say, affecting the affects of public audiences might service a subset of the goals associated with an infrastructure such as a sewerage system, say by ‘inciting’ or ‘nurturing’ some sort of general support for those who administer and manage, or else work ‘within’, the infrastructure. It will not have gone unnoticed that the terms deployed to address this constituency have been ‘audiences’ and ‘publics’ rather than the more commonsensical one of ‘users’. I did not do this to draw any hard and fast distinctions, but rather to indicate that these identities routinely blur into each other. The uses enabled by infrastructures are often entangled with the representations through which those infrastructures are enacted. The uses of such services as electricity, gas, water, sewerage, telecommunications – are enmeshed with many texts (bill inclusions, TV advertisements, etc.) that perform such things as commitment to price limitations, promises of future improvement, and notice of alternative service providers. There is a clear affective dimension to these texts: they seek to shape how users as audiences and publics experience and attach themselves to particular infrastructures (as consumers, as observers and as citizens). The case of the fatbergs is helpful because it can be used to examine this affective dimension in some detail: In particular, by tracing the *multiple* enactments of the fatbergs, and the *diverse* array of affects incited by these enactments, the process of infrastructuring as it is partially and complexly mediated through affect can be explored.

So far, I have used the notion of affect as if it were transparent. Needless to say, affect has been a topic of considerable discussion and debate (see, for example, Blackman and Venn, 2010; Clough and Halley, 2007; Gregg and Seigworth, 2010). In STS, affect is also playing an increasingly important conceptual role, having been used to examine processes ranging from scientific epistemic practice (e.g. Kerr and Garforth, 2016; Latimer and Lopez Gomez, 2019; Martin et al., 2015; Puig de la Bellacasa, 2011) through to public engagement with science and technology (e.g. Davies, 2014, 2019; Michael et al., 2018). While the version of affect that informs the discussion draws on these debates, it is especially inspired by the work of Anderson (2014).

Much of the recent writing on (the turn to) affect has drawn a divide between emotion broadly understood as a socialized, subjectively accessible state, and affect, broadly understood as an asocial, objective intensity within the body (e.g. Massumi, 2002; Thrift, 2007). There have been various critiques of this division, one of the most elaborated being that of Wetherell (2012), who persuasively argues that it is not clear what value such a division affords. As she writes: ‘It is a mistake to remove pre-conscious visceral perception from its usual and habitual world/brain/body/mind contexts, and to artificially freeze and isolate affect as a separate element from the dynamically integrated sequences in which these things normally operate. No easy distinction can be made between visceral and cultural meaning-making, and why should we make one – what is the advantage?’ (p. 67). Anderson (2014) makes a parallel point when he suggests that ‘we must pay attention to how representations function affectively and how affective life is imbued with representations. In addition, ideas about affect, or representations of affect, may take on an affective life of their own’ (p. 14). In other words, we cannot presume affect to be asocial.

Anderson defines affect in terms of ‘a body’s “capacity to affect and be affected”, where a body can in principle be anything’ (p. 9). He expands on this to note that it implies that affected and affective bodies emerge from and, in turn, mediate and shape a nexus of relations. Accordingly, affective life is necessarily mediated by specific, patterned material arrangements comprised of heterogeneous elements. This indicates that affects need always to be specified in their particularity as part of specific arrangements. From Anderson’s wide-ranging discussion, I point to two key forms that such arrangements might take: apparatuses and atmospheres. In the case of apparatuses, affective life is an ‘object-target’: affects are performed through specific means by which affects are named, rendered knowable, and opened up to intervention. In the case of atmospheres, these are enveloping affective characteristics or qualities that derive from ensembles of diverse elements (even if these can’t always be easily differentiated). These qualities are ambiguous in a number of ways. Atmospheres straddle the objective and subjective (we can find ourselves ‘within’ atmospheres, and we must attune ourselves to them). Atmospheres are also both situated and mobile or diffuse (e.g. an atmosphere can typify one group but also be felt by another). Further, atmospheres are at once present and absent, fully formed and in the process of formation. As Anderson notes, apparatuses and atmospheres are not in practice distinct: Apparatuses can be designed to engender atmospheres, and atmospheres can provide the conditions in which apparatuses become effective. For example, apparatuses for engendering anticipations about future technological prospects can also shape affective atmospheres of excitement and wonder, while the same atmospheres can attune audiences, publics or users so that they are more receptive to those apparatuses (see Stewart, 2011; Woolgar and Neyland, 2013).

To be sure, this is a highly truncated account of, and of course does not do justice to, Anderson’s rich discussion. Nevertheless, for present purposes, it does at the very least alert us to the ways in which infrastructuring can be embroiled in, and can entail, ensembles of elements. On the one hand, these ensembles imply discrete apparatuses that directly intervene in affect. On the other hand, they can engender affective atmospheres that envelop and affect audiences in more diffuse ways. Moreover, these apparatuses and atmospheres can be tightly interwoven (see below). Crucially, given that affects not only impact bodies, but are mediated by those bodies, those affected audiences can go on to affect infrastructures, indeed, become part of the process of infrastructuring (as mentioned above). Having made this point, it should be acknowledged that there is nothing inevitable or inexorable in the functioning of affective apparatuses or atmospheres. People can, in one way or another, resist or ignore apparatuses (e.g. Woolgar and Neyland, 2013) or fail to attune to atmospheres (see Brown et al., 2019).

By way of preview, in what follows, the analysis will touch on enactments of fatbergs that act in apparatus-like ways directly to target an audience’s specific affects (such as guilt of flushing wet wipes down the toilet). However, the main focus will be on affective atmospheres as mediated by various enactments of the fatbergs (for instance, the diffuse impact of multiple enactments of the fatberg that together envelop the audience in an affective atmosphere of amused, resigned identification – we are Londoners who can laugh at the recalcitrance of our sewerage system).

## Notes on method and analysis

My broad aims here are to examine the several ways in which the London fatbergs were enacted and how these entailed portrayals of London’s sewerage infrastructure, of London itself, and of the audiences of the fatberg (who, as noted above, might also be routine users of the infrastructure). My more specific objective is to explore how these enactments operate affectively, potentially shaping the affects of audiences. As this is a study of enactments of the fatberg, it is not possible to say anything definitive about the audiences’ affects: At the most, I aim to make a persuasive argument that the enactments of the fatbergs mediate atmospheres that ‘invite’ particular audience affects.

I collected empirical material through a snowballing method in which online newspaper reports served as an initial entrée into other forms of representation. Crucially these included TV reports and programmes (especially Channel 4’s *Fatberg Autopsy*), and the Museum of London’s *Fatberg!* exhibition. To a lesser extent, I also followed design engagement with fatbergs. Occasionally, I had fortuitous encounters with representations of the fatberg, such as a cartoon seen in London’s newspaper, the *Evening Standard*, or the closing statement of BBC Radio 4’s long-running comedy show, ‘I’m Sorry I Haven’t a Clue’.

I subjected the accumulated materials to a light semiotic and discourse analytic reading (e.g. Barthes, 1977; Potter and Wetherell, 1987), trying to draw out key themes related to the affective enactment of London fatbergs. On this score, my analysis has some similarities with other treatments that derived affects from various cultural artefacts. Particularly pertinent is the work of Oikkonen (2017; see also Linden, 2019, for a more granular approach) who provides an excellent analysis of the affects associated with the Zika epidemic as enacted through the pages of the *New York Times*. She focuses ‘on circulating phrases, recurring ways of speaking and narrating, and structural ways of organizing and framing the epidemic’ (p. 686). She strategically uses only text, in order to ‘ensure a clear analytical focus’ (p. 686); in addition, a key aim of her article is to ‘develop methods for textual analysis of affective dynamics’ (p. 684). As she states, her analysis could be enhanced with the inclusion of a broader range of materials. While Oikkonen generates insight into a nuanced and ambiguous complex of affective dynamics, here I aim to address a multiplicity of affects associated with the fatbergs, and how these affects are rendered through an ensemble of disparate elements (that together comprise an affective atmosphere or apparatus). Thus, in addition to texts, I also analyse images, displays, lighting, dialogue, etc. for their more or less concerted affective content. Part of the critical dimension of my analysis lies in the ways in which these enactments of the fatberg cumulatively blur a range of geographical, public and political differences, and, in the process, detract from the ways in which infrastructuring of the sewerage system could be done otherwise.

## A multiplicity of fatbergs and their affects

### An overview

The popular media circulate some basic features of fatbergs. They are masses of fat that have collected and solidified in sewers (mainly in cities in the developed world), in the process seriously affecting the flow of sewage. They can potentially lead to a backflow of sewage through toilets, baths and sinks. The first London fatberg was identified in 2013 in Kingston-upon-Thames, and the famous Whitechapel fatberg was discovered in 2017; it was the subject of the *Fatberg!* exhibition at the Museum of London. Another fatberg, three times the size of Whitechapel’s, was located in 2018 under the South Bank. This last one provided the material for Channel 4’s TV programme, *Fatberg Autopsy*. Inevitably, there is a Fatberg musical in the pipeline (Coughlan, 2018).

These standard media accounts affectively enact fatbergs in a number of ways. Much that has been said about fatbergs centres on their ‘disgustingness’. However, this is by no means the sole affect associated with fatbergs: in what follows, I discuss a limited selection of affects, though these affects are by no means mutually exclusive nor especially cogent. I identify five broad affects, along with the ways in which these are each mediated through the particular enactment of the fatbergs and their properties. These are brought under the following headings: mass and pride, chemicals and courage, physics and shame, microbes and anxiety, light and pleasure.

### Mass and pride

To draw on Douglas’s (1966) classic account of purity and danger, the fatberg is matter out of place. Or, rather, it is accumulated matter out of place. Or, better still, it is amassed matter that should have no place at all. Again and again – in newspaper and online articles, in the *Fatberg!* museum display, the *Fatberg Autopsy* TV programme – the fatberg is performed in terms of its massiveness. Indeed, the contemporary connotations of unhealthiness that attach to fat (Lupton, 2018), especially when bodily fat is accumulated beyond a prescribed, explicit or tacit, threshold, are echoed in this refrain over the massiveness of the fatbergs. This is reinforced by the fact that this fat is also seen to be indicative of too much oil in the diet – of eating badly and irresponsibly. It is partly because of its sheer size that the fatberg feeds into an affective atmosphere of sociomaterial unhealthiness of the city, whether that concerns the population and or the sewerage infrastructure.

By the same token, there is a sense that if London is going to display its unhealthiness by virtue of its hosting of fatbergs, it should be a peculiarly Londonesque unhealthiness. This is manifested in the routine measurement of the London fatbergs in terms of units that derive from, signify and arguably celebrate the uniqueness of London. This is captured in an article in the UK left-liberal broadsheet, *The Guardian*:

The units in which it was routinely measured gave away its birthplace. This being a London phenomenon it was invariably described in local currency: at 820 feet, the fatberg was ‘longer than Tower Bridge’ or ‘twice as long as Wembley Stadium’ and ‘the weight of 11 double-decker buses’. (Adams, 2018)

This adds an additional layer to the performance of London-ness: ‘London’ can be ironic about the iconicity of London (and its fatbergs) by humorously using its indigenous units to chart its own seeming infrastructural decline. This irony is underlined by a cartoon in London’s local newspaper *The Evening Standard* (5 June 2018). In the first frame, two trendy Londoners use a telescope to peer through a drain cover into the sewer, one saying, ‘I’m sure the FATBERG’S still down there, Izzy….’ In the second frame, the two main protagonists, along with a guide, are in a row boat, the guide saying, ‘You MIGHT see it if you’re lucky – there’s no guarantee.’ In the final frame, one of the protagonists holds up a camera phone, while the guide says, ‘It’s a bit like the Northern Lights, only smellier.’ Here, the fatberg is ironically compared to an elusive natural phenomenon worthy of touristic pursuit. Even if none of these things is realistic, the cartoon nevertheless plays with a double irony (Muecke, 1969) in which pride is enacted as the underside of ridicule.

It would appear that a degree of local London pride is being performed. There is an ownership of the Whitechapel fatberg (which at the time of writing has received the bulk of the publicity), an ownership that is reinforced by the almost peremptory references to fatbergs that have been detected elsewhere in the world. London pride is further expressed in the almost unseemly speed with which the fatberg went from sewerage problem to star exhibit at the Museum of London. Here, the fatberg became a mass that involves, or entangles, evidence that reveals London’s present history. As one of the information panels put it:

As the Museum of London, it’s important that we collect things that reflect the highs and lows of living in the city, today as well as in the past. We can tell a lot about a society by studying its waste. Many historic items in our collections were found in cesspits, or were never considered worth keeping.

Fatbergs are disgusting, fascinating things which mark a particular moment in London’s history. They are modern monsters, created by people and businesses who discard rubbish and fat which London’s Victorian sewer system was never designed to cope with. London is built on invisible, often ageing infrastructures that many of us take for granted and rarely think about. But the size and foulness of fatbergs make them impossible to ignore and remind us of our failings.

In addition to emphasizing the London-ness of the fatberg, this text also performs pride, this time with regard to the fact that the fatberg is being taken seriously as an archaeological artefact. It is not simply foul waste but a medium through which to derive insights into the life of contemporary London. The text thus serves in an affective atmosphere of disgust but also pride. Along the way, the text points to a number of other features of the fatberg – the ageing of infrastructures, the failings of people – that are taken for granted and even naturalized.

However, this text also serves as (part of) an apparatus insofar as it directly addresses the affects of Londoners, not least as it comments on their ‘failings’. It connects the discrete affect of shame to the fatberg as a serious object of study, and hints at the need for greater responsibility (and more responsible behaviour). In this regard, it reflects museums’ longstanding role in the shaping of public visitors (e.g. Heatherington, 2011; Macdonald, 1998, 2007).

### Chemicals and courage

Channel 4’s *Fatberg Autopsy* (first screened 24 April 2018) makes clear that the process of identifying, accessing, breaking up and removing the fatberg from the sewer is an immensely difficult one. When the programme’s ‘TV presenter and science enthusiast Rick Edwards’ enters the sewer, it seems that he can’t help retching from the sheer stench of the sewer. On top of this stench, workers have to deal with the discomfort of heavy-duty protective clothing and breathing equipment in cramped, difficult physical conditions. Especially challenging for the ‘flushers’ – the Thames Water employees tasked with ensuring that smooth sewage flows are sustained – is the physical resistance put up by the fat-laden sewage through which they wade. Adding to this is the danger of carbon monoxide and hydrogen sulphide poisoning: The flushers can work only when the concentration of these gases are below certain limits (they carry warning devices that are triggered when concentrations become excessive). Finally, there is the sheer physical exertion required to break up the fatbergs. While flushers can use high pressure hoses to break up some of the fatberg, sometimes it is too hard and they must attack it with shovels and picks. As one of the information panels at the *Fatberg!* exhibition states:

Fatbergs are rock-solid and removing them from London’s sewers is back-breaking work in cramped, filthy and dangerous conditions. High-powered jet hoses are needed to break them up, so they can be sucked out of the sewer by tankers. Some parts of the Whitechapel fatberg had to be hacked from the sewer walls with nothing but brute force and shovels. The fatberg took nine weeks to remove, with eight waste engineers working nine hours a day, seven days a week.

The sense of courage and exertion is redoubled by the ways in which the fatberg is characterized. As the following quotes from *Fatberg!* indicate, it is commonly represented as a monster, a beast or creature that needs to be defeated.

It’s a total monster … it’s like trying to break up concrete. (Thames Water’s Head of Waste Networks, Matt Rimmer)

Nailing the fatberg was like battling a giant Harry Potter movie creature beneath the streets of London. (Andy Brierley, Director of Lanes Utilities, maintenance partner for Thames Water’s waste networks)

Our work is finished, and the beast is finally defeated. (Alex Saunders, Sewer Network Manager, Thames Water)

If the London fatberg is a chemical nightmare, it is personified as a monster, which therefore necessitates extraordinary acts of courage, endurance and heroism. These enactments of London’s fatbergs contribute to an affective atmosphere in which the audience can revel in, be grateful for, and indulge admiration for the bravery of the sewerage professionals. However, the beastliness of the fatberg plays an additional role: It renders the fatberg a discrete entity bound up in a discrete event in a discrete setting. The motif seems to be a dramatic one of overcoming a singular challenge (albeit repeated across London’s sewerage system – monsters do not rest!). This distracts attention from the more routinized, mundane procedures of structural maintenance and blockage removal, and hence from the sewerage system as a process of infrastructuring. The beastliness of fatbergs engenders an affective atmosphere through which audiences are incited to sense the sewerage system as a pre-existing structure under attack from an extraneous source, rather than as an on-going process of maintenance and repair.^1^

### Physics and shame

Fatbergs accumulate and grow because fat can attach to something. That something, it turns out, is primarily wet wipes. Because these tend not to break down in the water flow of the sewers, wet wipes serve as physical surfaces to which fat can stick and accumulate, along with other objects (including condoms and tampons) which serve as further surfaces to which more fat can attach. However, the fat that accumulates loses its usual consistency (oily, semi-solid) because it combines with calcium in the water to undergo saponification. That is, it turns into soap. Nevertheless, its association and combination with the most intimate of artefacts (condoms, wet wipes, tampons) and its blockage of the free flow of human waste, and thus its entanglement with bodily excreta, together enact the disgustingness of the fatberg, and not its cleanliness. The fatberg enacts shame: the shame of lazily disposing oil and fat down the sink, the shame of having bodies that are involved with materials that clean corporeal waste and capture sexual effluvia.

As derived from the *Fatberg Autopsy* TV programme, this account of the various ingredients that comprise the fatberg is a-gendered. But, in the event ‘3 Days of Fat’, designer Lucy Sanderson (2019) notes that the fatberg is composed of particularly ‘feminine’ hygiene and cosmetic products, suggesting the fatberg might also be understood as a peculiarly gendered entity.

In *Fatberg Autopsy*, the blame is placed squarely on the wet wipe. This is underlined through an experiment in which flasks filled with water and different sorts of ‘wipe’ are agitated in a way that mimics the flow of water in a sewerage system. While toilet paper disintegrates readily, and some brands of ‘flushable’ (as advertised on the packaging) wet wipes break up reasonably well, other ‘flushable’ wipes fail to break up at all (not least because they incorporate the thermoplastic polymer polypropylene to increase strength).

The programme urges people to stop flushing wet wipes. Edwards says semi-jokingly to a member of the public at one point, ‘you′re responsible for the fatberg’. Arguably, through the TV vox pop (voice given to members of the public through journalistic interviews) we are witness to an affective apparatus. This operates as a version of ‘making’ (Hacking, 1986) citizens through the responsibilization of wet wipe disposal – the TV interviewee must acknowledge their guilt and thus change their behaviour (but on fat and oil waste disposal see Ibañez Martin and de Laet, 2017).

But the main suggestion is to alter the labelling on wet wipe packets so that ‘flushable’ means that wet wipes are ‘sewer friendly’, rather than simply capable of getting around a toilet’s u-bend. For those wet wipes that do not break up, their packets should prominently sport a ‘do not flush’ logo. As the voiceover notes, at that moment there was no regulation to force compliance on the part of manufacturers.

This view of the wet wipe has been reinforced in a *Guardian* article entitled: ‘From babies’ bums to fatbergs: How we fell out of love with wet wipes’ (Watts and Smithers, 2018)

(Fatberg) Blockages cost the UK about £100m every year, according to Water UK’s director of corporate affairs, Rae Stewart: ‘Water companies spend billions of pounds every year improving water and sewerage services in this country, but our sewers are just not designed to handle these new wipes which clog up the system. Sewer blockages end up costing the country about £100m every year so it’s clear that something needs to change.’

In all this, the fatberg is enacted as a composite involving numerous materials and compounds that are tied to shame (and guilt and responsibility), which need to be combatted through various changes in behaviour at both personal and corporate levels. As we have seen, central to the fatberg’s composition is the wet wipe. The necessity for wet wipes remains unquestioned: There is no mention of older technologies such as the flannel, for instance. Here, levels of cleanliness and practices of hygiene as mediated by wet wipe use stand unchallenged (see Shove, 2003). It is against this affective backdrop of wet wipe-related cleanliness that the affective atmosphere of shame and responsibility is enacted.

Can the wet wipe’s established and maintained sociomaterial routines of consumption and disposal themselves be thought in terms of a ‘soft’ infrastructure? That is to say, can the affects of shame and responsibility be understood as emergent from the clash of two infrastructures, the ‘softer’ wet wipe ‘system’ and the ‘harder’ sewerage system? While I will not pursue this in detail here, I note that an important aspect of the dynamism of infrastructuring is the (affective) relationalities with, and adaptability to, other infrastructurings. As various authors have suggested (e.g. Bollinger et al., 2014; Chappin and van der Lei, 2014), the complex interactions and intersections of different infrastucturings have received surprisingly limited social scientific attention.

### Microbes and anxiety

In addition to the anxieties that arise with the possibility of backflow of sewage into homes because of blockages caused by fatbergs, other risks have been identified. Thus, the *Fatberg Autopsy* TV programme displays biological investigations of the South Bank Fatberg. These reveal a range of bacteria that include E. coli, Listeria, Campylobactor, and antibiotic-resistant bacteria. This is narratively dramatized over the course of the documentary. The programme begins with the initial tropes of epidemics, plagues and (as in the Museum of London exhibit) monsters, and completes this narrative arc with the presenters’ expressions of horror at the reveal of antibiotic-resistant bacteria, and the scientists dire warnings that these germs could kill us all.

One potentially interesting aspect of this enactment is the surprise associated with the discovery of such colonies of microbes. The audience is presented with the fatbergs as a source of possible infections, with potentially catastrophic effects. Yet fat is, after all, a food, and it would be a surprise if there were not bacteria growing on (and other organisms feeding off) it. Also, it would be a surprise if there were no dangerous bacteria in sewerage systems; these microbes presumably pre-existed the fatberg and have merely exploited its presence.

In this performance of the fatberg, it would appear as if the fatberg has somehow ‘gathered’ and ‘concentrated’ the microbes and the risk. Indeed, the fatberg seems to reflect a breach in the sewerage infrastructure, allowing dangerous microbes to amass. In this respect, the enactment of bacterial threat and the associated affective atmosphere of anxiety rests on a tacit version of infrastructure under attack by an unruly nature. Underlying this enactment is what might be called a ‘anthropocentricity of infrastructuring’ in which infrastructures are seen simply as human-oriented structures at the service of human communities and populations. By contrast, as various authors have noted, infrastructuring is necessarily in the thrall of extended naturecultures (e.g. Haraway, 2003): nonhumans make use of infrastructures in all sorts of ways (e.g. the use by animals of road verges as ecological corridors – Michael, 2004).

Following Hinchliffe et al.’s (2012) analysis of typical approaches to zoonoses, microbes are enacted as external threats to a healthy society, rather than chronic components of, and intrinsic flows within and through, that society. As Hinchliffe and his colleagues might say, this othering of microbes serves to limit the range of practices and relations that make up infrastructuring. For these scholars a more topological formulation in which the human, nonhuman and the technological are entangled in multiple and changing ways is preferable, not least because it opens up new ways of developing more adaptive infrastructures. However, in the present case the surprise at the discovery of dangerous microbes with the fatberg evokes an affective atmosphere of anxiety linked to a version of infrastructure that is under attack.

### Light and pleasure

However, running alongside these various negative affects are other positive ones: there are pleasures to be had with the fatberg (over and above those entailed in the sense of London pride). Both the Museum of London display and *Fatberg Autopsy* deploy certain techniques to evoke the cinematic horror genre. In both, for example, lighting is arranged in ways that echo the darkness of the sewers and their partial illumination as the fatbergs are removed. In relation to the exhibit, pools of light illuminate quotes placed on the walls, cabinets of equipment, and the fatberg fragments in their own glass display cases. The movie horror genre, with its calculated patterning of fragmented and partial visibilities (Cherry, 2009) is being tacitly referenced here. In the case of *Fatberg Autopsy*, the space in which the autopsy takes place is housed in an old Victorian sewerage processing building. The lighting is arranged in order to optimize an atmosphere of unease. The light is at its strongest around the science section, where chemical and microbial analyses of the fatberg take place. The epistemic illumination provided by science can, however, be counter-posed with the evident horrified relish of the scientists and the presenter as they describe the danger-laden components of the fatberg. This can be tellingly contrasted with the deadpan responses of the flushers who, in breaking up the fatberg at their separate table, treat the removal of syringes, condoms and so on as a practical and mundane matter. Where the scientists and presenter enact danger and horror, the flushers do everyday life.

Of course, the horror genre also affords considerable pleasure; horror is to be enjoyed. For all the various terrors, dangers and dreads enacted with the fatbergs, these are safely mediated and contained through the recognizable markers of the horror film. The seemingly atmospheric affects of horror are thus diffused by the affects of pleasurable familiarity: The fatbergs (and their infrastructures) are simultaneously mysterious and uncontrolled, and known and domesticated.

In sum, the horror of the fatberg is at once emphasized and ironicized by the generic horror lighting. It is to be taken seriously but not too seriously. A public-cum-audience is enacted as concerned about, but also capable of distancing itself from, and smiling wryly at, the fatberg. Further, the public audience is given a glimpse of the (filmic) constructedness of the fatberg while also encouraged to engage with its supposedly unadorned reality. In Latour’s (2010) terminology, the fatberg can be said to have taken on the guise of a factish.

In the Museum of London exhibit, lighting also takes on a very different form. The illumination is brightest in the gift shop. There, under harsh lighting, is a display of fatberg-related products that one can buy: ‘Don’t Feed the Fatberg’ t-shirts and bags, depicting a white ghost-like fatberg with empty eyes and a gaping mouth climbing over a wall or out of a sewer, packets of ‘Fatberg Sludge’ fudge, and copies of the book *London’s Sewers*, to mention the most obvious. Wound around the table on which these products are arranged is black and yellow striped hazard tape. The direct and intense lighting allows one to see the products on sale, to take pleasure not least in the humour they provide. For visitors, there is the prospect of a pleasing juxtaposition of consumer pleasure and the filmic tingle of terror of the fatberg ‘itself’.

However, it is also possible to understand this particular enactment of the fatbergs in terms of the relation between consumption and affect. As I noted earlier, affect entails both being affected and being able to affect. Consumption as the purchase of souvenirs allows for the expression and sharing of experience, a means of creating affect for others by showing one’s mementoes (Lury, 1996). Better to see the fatberg ‘goods’ under sharp light is, in this case, better to be able to choose which product is most likely to enable such affective sharing. As such, in the setting of the brightly lit gift shop, it would seem that the affective atmosphere of pleasure and domestication of the fatberg (and its infrastructure) is reinforced, though this time through the prospects of consumption and sharing.

## Discussion

The feelings associated with fatbergs, while mainly negative, are not exclusively so. Indeed, crucially, they are multiple. As in Harvey’s (2012) analysis of the topological character of infrastructures, and Bowker et al.’s (2010) framing of infrastructure as inherently multiple, the fatberg is rendered in numerous ways that inform a plurality of affects. Such affects have ranged across pride in the ‘London-ness’ of the fatbergs, admiration at courage of the flushers, guilt at the flushing away of wet wipes, anxiety at the potential diseases facilitated by the fatbergs, and pleasure in the consumption of the fatberg as generic cinematic product and in the form of souvenir. In the process, the London’s sewerage infrastructure is enacted in various ways: as a place of microbial danger, as a marker of London’s renowned Victorian heritage (both infrastructural and cultural), as a partially effective conduit for the products of irresponsible behaviour, and as a site of hard work and bravery.

It would seem that the fatbergs as enacted through various affective atmospheres and apparatuses are indicative of an infrastructural breakdown. But let us take a step back here. In the case of the fatberg, we are witness to potential failure, to ongoing threat, but also to actual success. After all, the fatbergs at issue were identified and, albeit with a concerted effort, effectively removed. What seems clear is that, in keeping with the ‘mythical’ view of infrastructure (e.g. Bowker et al., 2010) as an entity (as opposed to a nexus of dynamic relations), London’s sewerage system is enacted, at least in part, as an object that is ‘separate’ from its continuing maintenance. Put otherwise, the affective enactments of the fatbergs have served in the dramatization of the sewerage infrastructure as a discrete entity.

And yet, in keeping with one of STS’s classical approaches to infrastructure, the focus on breakdown can serve as a means to tracing analytically the manner of an infrastructure’s operation (e.g. Star and Ruhleder, 1996). In the present case, we are faced less with a ‘de facto’ breakdown, or even a more experiential ‘affective rupture’ (Knox, 2017) but with mediated affective – dramatized – enactments of potential disruption. What do such enactments do, not least for our analysis of infrastructures? To be sure, such enactments map onto other ‘infrastructures’, not least those of the media and their news values. However, they also suggest that infrastructures, in their multiplicity, extend beyond the socio-material assemblages of technologies, operators, regulators, technologists, etc., to encompass users-publics-audiences. That is, part of the process of socio-material infrastructuring is the infrastructuring of public audiences, including their affective infrastructuring.

One example of this expansion of the notion of infrastructuring can be found in the work of Le Dantec and DiSalvo (2013; also Smedberg, 2019).^2^ These scholars advance the view that in the context of participatory design that is oriented toward infrastructuring, there is an attempt to identify and form attachments across ‘the social and material dependencies and commitments of the people involved’ (Le Dantec and DiSalvo, 2013: 242). This entails a ‘broadening (of) the view of what counts as innovation, moving away from a technocratic view of innovation toward one that includes social innovation: innovation that arises out of social interactions and action that arises from the constitution of a public …. (As such, there is a) federating (of) individuals in the discovery of unknown issues’ (p. 247). Infrastructuring applies to the prospective ‘users’ of a design innovation (part of which is the very nature of that innovation and the socio-material relations that enable its emergence). In the case of the fatbergs, there is less a participatory engagement with members of the public, and more a mediated one. Instead of La Dantec and DiSalvo’s users-cum-co-designers, the fatberg case entails users-cum-public audiences. Infrastructuring does not orient to emergent issues arrived at collaboratively, but to ‘issues’ enacted by experts and media practitioners. The infrastructuring thus concerns the making of publics affectively aligned with those issues (whether these be negative such as microbial risks, or positive such as the pride in London-ness). In essence then, I use ‘affective infrastructuring’ to denote the ways in which users, audiences and publics, through affective enactments, are constituted as elements within the process of extended infrastructuring that encompasses public audiences.

I propose two further analytic points derived from considering the cumulative effect of the various affects I have discussed. Instead of treating these multiple enactments of the fatberg and the affective atmospheres associated with them separately, is it possible to detect cumulative affective atmospheres that draw upon and aggregate these more specific affective atmospheres? Put otherwise, are there affective elements that are common across the affective enactments of mass and pride, chemicals and courage, physics and shame, microbes and anxiety, and light and pleasure? To be sure, the points that follow are more speculative, derived as they are from a reading of the cumulative impact of the affective enactments of the fatbergs, their infrastructures and their public audiences. Nevertheless, the hope is that they open up a discussion around the ways in which processes of affective infrastructuring might combine at several levels and across seemingly discrete settings.

First, across the various enactments of the fatbergs there seem to be manifestations of London in the abstract. Although the fatbergs are situated in particular sections of the sewerage system, they are presented as if their impact is on London as a unified object. Further, these impacts are generally portrayed as the results of the actions of London’s population as an undifferentiated ensemble. Other examples of this uniforming of London and its population are evidenced in the *Fatberg!* Exhibition, where the fatberg is the result of Londoners’ habits as they challenge London’s ailing sewerage system as a whole, and in *Fatberg Autopsy*, where the fatberg’s bacteria put all Londoners at risk.

This uniforming of London is thrown into relief when contrasted against the rare instances where London is not portrayed as a singular space. At one point in *Fatberg Autopsy*, the presenter jokes with a flusher about a better quality of fatberg in Kensington. Given Kensington’s wealthy residents, the presenter and flusher joke that some local fatbergs will contain more expensive goose fat than others. At this moment, London is not singular but variable in terms of wealth. This is a contrast echoed in the ′Smell of the City′ design project developed by Victoria Jones (2017). Jones produced fatberg lumps apparently drawn from – though in actuality artificially treated to signal olfactorily – different parts of a city′s sewerage system. In the related exhibition, visitors smelled these lumps and were subsequently interviewed as a way of exploring the relation of smell to memory and locality. Clearly this suggests that the fatberg is potentially a marker of class and cultural differentiations across the city – which are elsewhere usually effaced. On this score, the media portrayals of fatbergs reinforce a sense of collective London identity that detracts from inequalities and differentiations. To the extent that an affective sense of communitas is conjured, the London sewerage system becomes ‘our’ sewerage system. Together, the multiple forms of infrastructuring enact a common or uniform public that affectively aligns with a common or uniform infrastructure.

Second, there is a similar affective aggregation in relation to the temporality of the fatbergs and their infrastructure. In the Museum of London′s stated rationale for building an exhibit around the Whitechapel fatberg, in various media reports, in the *Fatberg Autopsy* documentary, London’s sewerage infrastructure is portrayed as ageing, as if this were something that is natural or inevitable. This temporal naturalization, it can be tentatively proposed, underpins a cumulative affective atmosphere of resignation, or acceptance. This is what infrastructures do – they necessarily age, and Londoners should accommodate to this.

Like the unity of London, this is thrown into relief when contrasted with an alternative enactment. In one newspaper article, there is a suggestion that fat can actually be captured in fat traps that can be introduced to, integrated into, and maintained within, sewerage systems (Moss, 2018). It is claimed that some cities, whose water utilities are publicly owned, have these traps cleared out more assiduously than those cities whose utilities are privately owned (as is the case in London and Thames Water). Whatever level of credence one wants to attach to this claim, it is nevertheless a critical reminder that these infrastructures are parts of governmental and corporate assemblages that shape what is considered doable and that affect the forms that infrastructuring can take. Thus, from a critical perspective, infrastructures can be said to age because of lack of investment, or deficiencies in an appropriately attentive infrastructuring. In the enactments treated above, the common emphasis on infrastructure as an ageing entity, as opposed to as a continuous relational process, is central: The infrastructure declines of its own accord, rather than because a nexus of infrastructural relations and processes have been allowed to degrade. The affective atmosphere that emerges around these accumulating enactments is, as cautiously suggested above, one of resignation or acceptance.

## Concluding remarks

The multiple versions of London’s fatbergs have been associated with particular affects that serve in the attempted ‘making’ of public audiences (and their affective relations to the sewerage infrastructure). The term I tentatively coined for this is ‘affective infrastructuring’ which highlights how public audiences, through various affective enactments and mediated by atmospheres and apparatuses, are components in the process of infrastructuring.

This affective infrastructuring, despite the varying forms it takes, has the cumulative effect of rendering uniform the spatialization of, and enacting a naturalized temporality for, London’s sewerage infrastructure. The analysis I have presented here is specific to this case, not only because of the usual interpretative complexities, but also because of the socio-material complexities of the infrastructures themselves.

These circumspections aside, does the present notion of affective infrastructuring have any mileage beyond the specificities of the London sewerage system and its fatberg problems? Even for sewerage infrastructures, we might ask whether the affective enactments of fatbergs are peculiar to London, and if so, why this might be the case (for instance, is the London-ness of the fatbergs another version of the retrenchment of Britishness that is evidenced in debates around Brexit?). Would there be similar reactions to a fatberg problem in New York or Amsterdam? Further, would analogous affects be enacted if parallel problems arose in other infrastructures, say in transport or power systems? What additional affective apparatuses or atmospheres would be available?

The role of affect in infrastructuring explored here could be developed further, not least by elaborating the concepts of affective atmospheres and apparatuses. As I used them here, these at best connote the ends of a spectrum of specificity and targeting. However, the mediation of affective infrastructuring can no doubt take on other qualities, say, of longevity or intensity. We can see that the forms of ‘affect’ in affective infrastructuring would benefit from additional unpacking. But then so, too, would the ‘infrastructuring’ element of affective infrastructuring. As noted, any particular infrastructuring operates in relation to other infrastructurings – systems intersect in numerous ways. The fatberg was ‘in’ the sewerage infrastructure, but was also ‘circulated’ through media systems. Indeed, this raises the issue of where the boundaries of an infrastructure lie. Are enactments of the fatberg through various media outlets, and the telecommunications infrastructure that supports them, also ‘part of’ the infrastructuring of London’s sewerage system? Does the ‘soft’ infrastructure of cleanliness and hygiene, via the enactment of the fatberg and the wet wipe, blur or reconfigure the boundaries of the sewerage infrastructure? Under what circumstances do or don’t such infrastructural intersections take place? In any case, if the idea of affective infrastructuring has a value, perhaps it lies in the sorts of new questions it might prompt about the how to think infrastructures and infrastructuring.

## Notes

1. Speculative designers Mike Thompson and Arne Hendriks (2019) question the beastliness or monster-status of the fatberg. Instead of a terrifying otherness, Thompson and Hendriks propose an otherness that opens up possibilities for a series of interesting questions. In exploring these possibilities, these designers are gradually accumulating and congealing fat to build a fatberg that they intend to tow out into the North Sea. As they put it on their website: Looking out towards the ocean, FATBERG is easily spotted by the flock of seagulls circling above, swooping down to take a welcome nibble of its vast store of energy. Only a few square meters of the manmade island lift themselves above the waves, bobbing slowly up and down, side to side. If you ask the creators of FATBERG why they made it, you might hear: we feel the need to archive our times; or, we’re building a strategic reserve, anticipating difficult times ahead. The truth is they don’t know why, they simply know they must …. Their aim is not to define the issues raised by the fatberg, but to use its otherness as a means of examining the multiple and complex meanings and implications of fat but also infrastructures. In the present discussion, the considerable effort that has gone into making their fatberg is indicative of the multiple and complex conditions under which fatbergs can arise in sewerage systems (Thompson and Hendriks, 2019).

2. I encountered Smedberg’s (2019) piece, which involves a parallel use of the term ‘affective infrastructuring’, in the process of re-drafting this article. As in the critical participatory design ethos found in Le Dantec and DiSalvo’s (2013) work, Smedberg emphasizes the affective engagement of citizens in the municipal design of an ‘infrastructure’ (a neighbourhood) and new participatory ways of working across actors (citizens, designers, civil servants, architects). In contrast, the present paper uses ‘affective infrastructuring’ to denote the ways in which, through affective enactments, users, audiences and publics are constituted as elements in the process of infrastructuring.
